# High starchy food intake may increase the risk of adverse pregnancy outcomes: a nested case-control study in the Shaanxi province of Northwestern China

**DOI:** 10.1186/s12884-019-2524-z

**Published:** 2019-10-21

**Authors:** Liyan Huang, Li Shang, Wenfang Yang, Danyang Li, Cuifang Qi, Juan Xin, Shanshan Wang, Liren Yang, Lingxia Zeng, Mei Chun Chung

**Affiliations:** 1 Department of Obstetrics and Gynecology, Maternal & Child Health Center, The First Affiliated Hospital of Xi’an Jiaotong University, No. 277, Yanta West Road, Xi’an, Shaanxi Province 710061 People’s Republic of China; 2 Department of Public Health and Community Medicine, Tufts University School of Medicine, MA Boston, USA; 3 Department of Women’s and Children’s Health, Karolinska Institutet, Solna, Stockholm Sweden; 4 Department of Epidemiology and Biostatistics, School of Public Health, Xi’an Jiaotong University Health Science Center, Xi’an, Shaanxi People’s Republic of China

**Keywords:** Periconceptional dietary patterns, Starchy food, Adverse pregnancy outcomes, Factor analysis, Nested case-control study, Chinese women

## Abstract

**Background:**

There was a wider disparity in the diet characterization among most studies on diet and pregnancy outcomes in different countries, and the research in northern China is limited. Therefore, the purpose of the present study that was conducted in northwest China was to understand the dietary characteristics of periconceptional women and to explore the relationship between and specific dietary patterns with adverse pregnancy outcomes.

**Methods:**

A nested case-control study was conducted from October 2017 to November 2018 in Shaanxi, China. Based on a prospective cohort of 368 women who were pregnant or prepared for pregnancy, 63 participants who developed the outcomes of gestational hypertension, gestational diabetes, preterm birth, low birth weight, and birth defects were included in the case group. A total of 237 healthy pregnant women were included during the same period in the control group. Dietary intake was assessed using a validated food frequency questionnaire for the three months before pregnancy and the first trimester. Information on delivery details and antenatal pregnancy complications was obtained from the hospital maternity records. Dietary patterns were derived using factor analysis. Stratified analysis was performed on the overall, single and multiple adverse pregnancy outcomes categories. Adjustment was made for sociodemographic characteristics and nutritional supplement status.

**Results:**

Six major dietary patterns were identified. The ‘starchy’ dietary pattern, composed of high intake in noodle and flour products and/or rice and its products, was associated with the odds of developing of adverse pregnancy outcomes (OR: 2.324, 95% CI: 1.293–4.178). This risk remained significant following adjustment for potential confounders of maternal demographic characteristics and nutritional status (aOR: 2.337, 95% CI:1.253–4.331). Strong association were found during the first trimester of pregnancy, but showed no association during the three months before pregnancy (aOR:1.473, 95% CI: 0.682–3.234).

**Conclusions:**

High starchy food intake was associated with adverse pregnancy outcomes, particularly during the first trimester of pregnancy. Health education focusing on periconceptional dietary patterns could be a practical strategy for preventing adverse pregnancy outcomes.

## Introduction

Recent epidemiological evidence of an association between poor fetal growth and adult disease highlights the need to reconsider the influences which act on the fetus, and the role maternal nutrition may play [1]. From a prevention perspective, epidemiological research into the relationship between diet and pregnancy outcomes is essential. The use of dietary patterns is considered an estimation of the overall dietary habits of a subject and has become widespread in nutrition research as an alternative approach to study individual components of the diet [2–7]. The investigation of the association between different dietary structures and pregnancy outcomes has shown that specific dietary constituents can exert high risk or protection with regard to preterm birth [2, 3], shorter birth length [2], hypertensive disorders in pregnancy (HDP) [4], gestational diabetes mellitus (GDM) [5, 6] and small for gestational age infants (SGA) [7]. However, most of the results of these studies are inconsistent because of the cultural differences among various geographical regions and countries result in distinct dietary sources. There was a wider disparity in the diet characterization among most studies on diet and pregnancy outcomes in different countries [8]. For example, Chinese traditional staple foods are mainly rice and flour products based starchy foods. Although the components of starchy foods are included in some specific dietary patterns in some researches, the extraction of starchy foods with proven healthy foods such as vegetables/fruits [9–11], or with proven non-healthy foods such as fat /deep fried food [12] as a dietary structure may mask or exaggerate their effect. It raises the need for additional studies on specific dietary patterns to provide evidence on potentially preventative interventions to reduce the burden of adverse pregnancy outcomes.

Concomitantly, dietary patterns and adverse pregnancy outcomes are also associated with sociodemographic characteristics, such as family income, urbanization, and individual education [13, 14]. However, at present, the associations between the periconceptional dietary structure and adverse pregnancy outcomes in northern China have not been examined in detail. Also, there is a close relationship between periconceptional nutritional supplementation and pregnancy outcomes [15], but this important confounding factor is still often ignored in many studies.

We therefore analyzed the observational data collected for a nested case-control study in the northwest of China, to understand the dietary characteristics of periconceptional women and to explore the relationship between specific dietary pattern and adverse pregnancy outcomes based on the adjustment of nutrient intake and other important confounding factors.

## Material and methods

### Study population

We conducted a case-control study nested in a cohort. The cohort conducted from October 2017 in the First Affiliated Hospital of the Xi’an Jiaotong University, which encompassed a population of 368, to evaluate the associations between maternal environmental exposure during the periconceptional period and the cognitive development of offspring. The subjects were randomly selected from the women who were admitted to the hospital for pre-pregnancy examination or those who were already pregnant and within their 13^+ 6^ weeks’ gestation period. The medical record number, telephone number and home address of each participant were recorded at the time of recruitment to facilitate follow-up.

### Case control selection

The nested case-control was conducted on the impact of periconceptional dietary patterns on adverse pregnancy outcomes between October 2017 to November 2018. We excluded women who were not pregnant within three months (*n* = 21), women with abnormal pre-pregnancy examinations(*n* = 23), including gynecological inflammation, polycystic ovaries, tumors, anemia, hypertension, diabetes, and metabolic diseases, such as thyroid dysfunction. In addition, women with a history of genetic defects in birth (*n* = 1) and women who had been pregnant through assisted reproduction were also excluded (*n* = 1). The follow-up method was mainly used to track the prenatal and postnatal examination results recorded by the hospital maternity records, and the women who were transferred to other hospitals were followed-up by phone. During follow-up, subjects with the following five adverse outcomes that most common in northwest China [16] were included in the case group: gestational hypertension, gestational diabetes, preterm birth, low birth weight and birth defects. After excluding subjects who were stillborn and other outcomes (*n* = 6), lost to follow-up (*n* = 11) and nonresponse (*n* = 5) during the investigation, 63 participants were included in the case group. The remaining 237 healthy pregnant women recruited in the same period were included in the control group. The ratio between the case and the control groups was approximately 1:4. The women in the case group were divided into single and multiple adverse pregnancy outcome subgroups according to whether they had two or more adverse pregnancy outcomes. (Additional file 2: Fig. S1).

The blood pressure of pregnant women was monitored and recorded in each regular prenatal examination. The gestational hypertension was defined a systolic blood pressure ≥ 140 mmHg and/or diastolic blood pressure ≥ 90 mmHg on two occasions 4 h apart, developing after 20 weeks of gestation in a previously normotensive case in the absence of significant proteinuria [17]. Gestational diabetes was defined by the following cutoff endpoints according to the Chinese guidelines on obstetrics and gynecology: fasting plasma glucose concentration ≥ 5.1 mmol/l, and a plasma glucose concentration at 1 and 2 h after oral administration of 75 g oral glucose that was ≥10.0 mmol/l and ≥ 8.5 mmol/l, as determined by the oral glucose tolerance test (OGTT) instructions [18]. The results of the screening method for gestational diabetes were followed up after the 24 to 28 weeks’ period of prenatal examination. Preterm birth was defined as delivery between 28 and 37 weeks of pregnancy. Low birth weight was defined as a neonate weighing less than 2500 g within 72 h of birth. Birth defects refer to the physical structure and function abnormalities that occur before birth, including congenital malformations, chromosomal abnormalities, genetic metabolic diseases and functional abnormalities.

### Exposure assessment

Dietary information was collected at baseline through a 19-item validated food-frequency questionnaire (FFQ), these have been shown to represent the daily diet of Chinese women adequately [19] (details see Additional file 1). All participants were investigated and questioned twice by trained investigators via face-to-face interviews when they are recruited (collected the information during three months before pregnancy) and during their 13–16^+ 6^ weeks’ prenatal examination (collected the information during the first trimester of pregnancy). The FFQ included noodles and flour products, rice and their products, fruits and vegetables, meat, dairy products, beverages and fried food. For the majority of the food types, the participants provided information concerning how often they were consuming each food type according to the three months before pregnancy and the first trimester of gestation. The following options were used: 1) more than once a day; 2) 3–6 times a week; 3) 1–2 times a week; 4) 2–3 times a month; 5) less than once a month and 6) never or rarely.

### Covariate assessment

The baseline questionnaire inquired information about socio-demographic (age, residence, economic situation, occupation, education), smoking, alcohol intake and nutrient supplementation variables. The survey of nutrient supplementation was conducted at the same time as the dietary intake survey, that mainly included the types of nutrient and the supplemental dosage and duration during the three months before pregnancy and/or the first trimester of pregnancy, respectively. According to the type of nutrients that pregnant women consumed in addition to folic acid, they were divided into groups that received pure folic acid tablets, multivitamin tablets containing folic acid and other vitamins. The supplement dose was defined as a large dose supplement and a small dose supplement depending on whether the daily dose of folic acid was more than 0.4 mg. Similarly, the supplement dose was defined as long-term and short-term supplementation according to whether the duration of nutrient supplementation exceeded 90 days. In addition, we also recorded the pre-pregnancy BMI of pregnant women to assess their basic preliminary nutritional status, and we defined specific BMI levels of < 18.5 kg/m^2^, 18.5 kg/m^2^ to 24.0 kg/m^2^ and > 24.0 kg/m^2^ as emaciation, normal and overweight and obesity, respectively.

### Statistical analysis

All data were coded and assigned the following data cleaning and quality check. Epidata3.1 was used for data entry and logic error detection. Quantitative data were described as mean ± standard deviation and categorical data were presented by the composition ratio.

In order to describe the dietary patterns of the subjects, factor analysis with varimax rotation was performed to calculate the components on the 19 standardized food items. This analysis was based on the principal component method. The number of components that described this information was selected on the basts of the screen plot and the interpretability of the factor loading [20]. Food items with factor loading (absolute value) above 0.3 on a component were considered to have a significant association with that component. The explained variance for the individual components was subjected to rotation redistribution to achieve a simpler structure. The factor scores were computed for each woman by summing the food items consumed according to their factor loadings. According to the quartile of factor score, the 1st and 4th were divided into low intake and high intake subgroups.

The associations [odds ratios (ORs) and 95% confidence intervals (CIs)] between maternal dietary patterns and nutritional status, and adverse pregnancy outcomes were examined by logistic regression analysis. Possible confounding factors that were suggested by sociodemographic characteristics and nutritional supplement status were included as covariates in the multivariate logistic regression analysis for the dietary patterns. In addition, all analyses were stratified by overall, single, and multiple adverse pregnancy outcome subgroups.

All data were analyzed by the SPSS 18.0 software and the effect estimates were considered to be significant if the *p*-value was < 0.05.

## Results

A total of 300 pregnant women were included in the nested case-control study, with an average age of 29.75 ± 3.854 years. These were divided to 237 in the control group and 63 in the case group. Of the 63 patients in the case group, 35 (55.6%) women were single adverse pregnancy outcome, and 28 (44.4%) women were multiple adverse pregnancy outcomes (two or more).

### Association between sociodemographic characteristics and adverse pregnancy outcomes

The noted covariates of sociodemographic characteristics were age and residence, economic situation, smoking and alcohol intake (Table 1). From Table 2, we found that the risk of adverse pregnancy outcomes in subjects with age higher than 35 years old and was significantly increased (OR: 4.228, 95% CI: 1.762–10.149). In addition, the women who lived in the countryside (OR: 2.467, 95% CI: 1.025–5.937), and with a history of alcohol intake (OR: 6.724, 95% CI: 1.562–28.952) and smoking (OR: 62.142, 95% CI: 8.931–432.397) during the periconceptional pregnancy were also associated with increased risk of adverse pregnancy outcomes. Following multivariate analysis, only maternal age, smoking and alcohol intake were significantly associated with adverse pregnancy outcomes.

**Table 1:** Equilibrium test of demographic characteristics between the case and control groups

	Total	Overall adverse pregnancy outcomes		χ^2^	P
	N (%)	Case(*N* = 63)	Control(*N* = 237)		
Age					
≤ 25	25 (8.3)	1 (1.6)	24 (10.1)	16.957	0.001*
26–30	173 (57.7)	31 (49.2)	142 (59.9)		
31–35	77 (25.7)	19 (30.2)	58 (24.5)		
> 35	25 (8.3)	12 (19.0)	13 (5.5)		
Residence					
suburb/rural	276 (92.0)	54 (85.7)	222 (93.7)	4.281	0.041*
city	24 (8.0)	9 (14.3)	15 (6.3)		
Education					
junior high school or below	19 (6.3)	12 (11.1)	12 (5.1)	3.176	0.204
senior high school or graduate	126 (42.0)	102 (38.1)	102 (43.0)		
undergraduate or above	155 (50.7)	123 (50.8)	123 (51.9)		
Economic situation ^a^ (per month)					
poor level	33 (10.7)	10 (15.9)	23 (11.0)	1.162	0.559
moderate level	217 (73.7)	44 (69.8)	173 (73.0)		
good level	50 (15.6)	9 (14.3)	34 (16.0)		
Occupation					
peasants	19 (6.3)	6 (9.5)	13 (5.5)	1.368	0.242
others	281 (93.7)	57 (90.5)	224 (94.5)		
Smoking					
yes	5 (1.67)	3 (4.8)	2 (0.8)	4.662	0.031*
no	295 (98.3)	60 (95.2)	235 (99.2)		
Alcohol intake					
yes	8 (2.7)	5 (7.9)	3 (1.3)	8.553	0.003*
no	292 (97.3)	58 (92.1)	234 (98.7)		

^a^ The poor, moderate and good levels of economic status were defined as the monthly household income per capita were less than or equal to 4000 RMB (581.3 dollars), 4001 to 12,000 RMB (581.4 to 1744 dollars) and more than 12000RMB (1744 dollars), respectively

* *p* < 0.05

**Table 2:** The association between basic characteristics and adverse pregnancy outcomes

	Overall adverse pregnancy outcomes		
	N (%)	OR(95% CI)	OR(95% CI)^a^
Age			
≤ 25	1 (4.0)	0.19 (0.025,1.465)	0.184 (0.023, 1.485)
26–30	31 (17.9)	1.000	1.000
31–35	19 (24.7)	1.501 (0.785, 2.867)	1.438 (0.712, 2.902)
> 35	12 (48.0)	4.228 (1.762,10.149)*	4.743 (1.786,12.599)*
Residence			
suburb/rural	54 (19.6)	1.000	1.000
city	9 (37.5)	2.467 (1.025, 5.937) *	2.192 (0.682, 7.041)
Education			
junior high school or below	7 (36.8)	2.474 (0.896, 6.835)	0.671 (0.153, 2.940)
senior high schoolor graduate	24 (19.0)	0.998 (0.547, 1.821)	0.863 (0.434, 1.716)
undergraduate or above	29 (19.1)	1.000	1.000
Economic situation (per month)			
poor level	8 (25.8)	1.075 (0.312, 9.694)	1.153 (0.322, 4.123)
moderate level	41 (19.2)	0.733 (0.342, 1.567)	0.827 (0.362, 1.887)
good level	11 (24.4)	1.000	1.000
Occupation			
peasants	6 (31.6)	1.814 (0.661, 4.980)	1.493 (0.375, 5.945)
others	57 (20.3)	1.000	1.000
Smoking			
yes	3 (60.0)	62.142 (8.931,432.397)*	59.201 (6.877,369.251)*
no	7 (2.37)	1.000	1.000
Alcohol intake			
yes	5 (62.5)	6.724 (1.562, 28.952) *	6.583 (1.337, 26.589) *
no	3 (1.03)	1.000	1.000

^a^ Multivariate regression analysis and adjustment of age and residence, economic situation, smoking and alcohol intake

* *p* < 0.05

### Association between nutritional status and adverse pregnancy outcomes

From Table 3, the noted covariates were pre-pregnancy BMI and the nutrient supplementation during the first trimester. It can be deduced that pre-pregnancy overweight or obesity were significantly associated with adverse pregnancy outcomes (OR: 2.927, 95% CI: 1.534–5.587). Positive associations were also noted between pregnant women with short-term and low dose nutrient supplementation and adverse pregnancy outcomes, particularly during the first trimester of pregnancy (OR: 2.885, 95% CI: 1.262–6.593). The results from the adjusted model indicated that only pre-pregnancy BMI was noted, whereas the adjusted OR values decreased slightly to 2.412 (95% CI: 1.143–5.089).

**Table 3:** The association between nutritional status and nutrient supplementation and adverse pregnancy outcomes

	Total	Overall adverse pregnancy outcomes		
	N (%)	N (%)	OR(95%CI)	OR(95%CI)^a^
Pre-pregnancy BMI				
emaciation	44 (14.9)	6 (13.6)	0.735 (0.289,1.873)	0.699 (0.247,1.982)
normal	198 (66.2)	35 (17.7)	1.000	1.000
overweight or obesity	57 (19.01)	22 (38.6)	2.927 (1.534,5.587)*	2.412 (1.143,5.089)*
Nutrient supplement before pregnancy				
short term and small dose	205 (69.0)	43 (21.0)	1.150 (0.314, 4.219)	0.815 (0.173,3.836)
short term but large dose	12 (4.0)	2 (16.7)	0.867 (0.121, 6.215)	1.402 (0.160,12.25)
long term but small dose	64 (21.6)	14 (21.9)	1.213 (0.303, 4.863)	1.049 (0.206,5.351)
long term and large dose	16 (5.4)	3 (18.8)	1.000	1.000
Nutrient supplement of first trimester				
short term and small dose	57 (19.4)	18 (31.6)	2.885 (1.262,6.593)*	2.557 (0.810,8.068)
short term but large dose	37 (12.6)	6 (16.2)	1.210 (0.417, 3.511)	1.496 (0.480,4.664)
long term but small dose	113 (38.5)	25 (22.1)	1.776 (0.835, 3.774)	2.009 (0.672,6.006)
long term and large dose	87 (29.6)	12 (13.8)	1.000	1.000
Types of nutrients				
pure folic acid	164 (56.3)	40 (24.4)	1.618 (0.904, 2.933)	1.092 (0.417,2.863)
multivitamin with folic acid	127 (43.7)	21 (16.5)	1.000	1.000

^a^ Multivariate analysis and adjustment of age and residence, economic situation, smoking and alcohol intake

^*^ *p* < 0.05

### Dietary pattern identification

The KMO and Bartlett sphere tests were performed for the dietary frequencies of 19 food items during the three months before pregnancy and first trimester of pregnancy, and the statistics were 0.707 and 0.680, respectively. The Bartlett’s spherical test suggested a nonvalid independent hypothesis test. Six dietary patterns were selected to best describe the dietary patterns of the women after factor analysis. The variability of these patterns for the period of three months before pregnancy and the first trimester of pregnancy was estimated to 53.690 and 54.273%, respectively as determined by these six patterns.

The factor loadings obtained from the factor analysis are shown in Additional file 3: Table S1 and Additional file 4: Table S2. The first pattern was described as ‘animal protein’ due to the high loadings of poultry, beef and mutton, fish and shrimp, and pork. The second pattern yielded a dietary pattern high in the consumption of green tea, coffee and cola, which was denoted as ‘caffeine’. The third pattern was characterized by high intake of food with high-quality protein contents such as beans and their products, milk and its products, nuts and eggs, and was therefore classified as ‘healthy’. The fourth pattern was labeled ‘processed’ as the predominant foods with high loadings were processed or high-fat foods, such as pickles/sauerkraut, fried food, animal organs and garlic. The fifth pattern with high loadings was composed of noodle and flour products, rice and its products, and was thus labeled ‘starchy’. Finally, the sixth ‘vegetarian’ pattern comprised high contents of fresh vegetables and fruits.

### Association between dietary patterns and adverse pregnancy outcomes

**Fig. 1:**
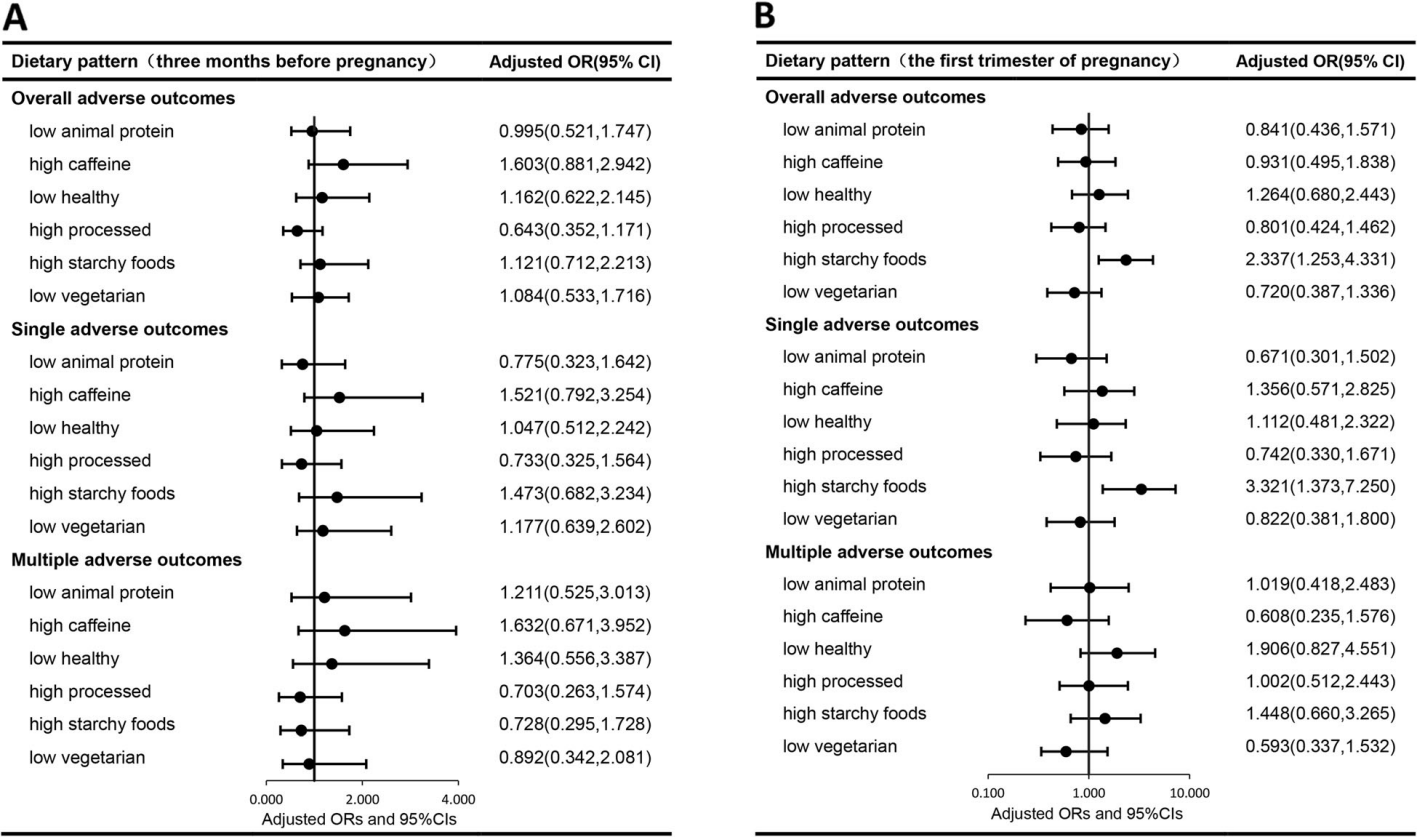
Associations between dietary patterns and adverse pregnancy outcomes during the three months before pregnancy (**a**) and the first trimester of pregnancy (**b**). The values presented are adjusted ORs (aOR) with 95% CIs and corresponded to the results derived from the multivariate analysis. The analysis was based on the noted covariates which were indicated in Tables 1 and 2. ORs adjusted for age, residence, economic situation, smoking and alcohol intake, and pre-pregnancy BMI. For the first trimester of pregnancy (**b**), ORs were additionally adjusted the nutrient supplementation

Univariate analysis (Additional file 5: Table S3) demonstrated that the pregnant women in the case group exhibited more ‘caffeine’ and ‘starchy’ food diets in the three months before pregnancy and more ‘animal protein’ and ‘starchy’ diets in the first trimester of pregnancy. Among them, the high intake of ‘starchy’ foods in early pregnancy significantly increased the risk of overall adverse pregnancy outcomes (OR: 2.324, 95% CI: 1.293–4.178) and the risk of single adverse pregnancy outcomes (OR: 3.307, 95% CI: 1.479–7.395). After adjusting noted covariates that were suggested by sociodemographic characteristics and nutritional supplement status, the multivariate analysis did not alter the significant association between the high intake of ‘starchy’ foods with the overall and single adverse pregnancy outcomes. The increased OR rates of 2.337(95% CI:1.253–4.331) and 3.321 (95% CI:1.373–7.250) were retained for both of these analyses, respectively (Fig. 1, Additional file 5: Table S3). No significant association was noted between other components and pregnancy outcomes.

## Discussion

### Main findings

In this nested case-control study, we identified an association between ‘starchy’ dietary pattern (characterized by high intake in noodle and flour products and/or rice and its products) and adverse pregnancy outcomes. Strong association were found during the first trimester of pregnancy but showed no association during the three months before pregnancy.

### Interpretation

We found that the dietary structure of China was mainly characterized by the “plant-animal balanced” pattern, which high in the consumption of grains, protein contents such as beans and their products, milk and its products and comprised high contents of poultry meat. It is similar to Japan which is also an Asian country [21]. However, it is quite different from the ‘plant-based’ dietary structure of India and Pakistan [22], the ‘animal-based’ dietary pattern of European and American countries [23], and the ‘Mediterranean’ dietary structure of Italy, Greece (comprised high contents of deep-sea fish, olive oil and fresh fruits) [24].

Besides, our results further demonstrated that high-starchy dietary patterns were associated with adverse pregnancy outcomes, but the existing researches on starchy foods were limited [14, 15, 25, 26]. Similar to our findings, Okubo et al. found that the maternal dietary pattern of excessive intake of ‘wheat products’ was more prone to nutritional imbalance than the high-protein dietary maternal pattern, resulting in a higher tendency to lower birth weight and the incidence of small for gestational age infants [27]. A research in Colorado also found a link that starchy foods such as potatoes, rice and other starchy vegetables, were associated with higher fasting glucose and greater newborn adiposity. These findings were also consistent with previous studies reporting inverse associations between the intake of dietary whole grains and insulin resistance [28, 29]. However, our results were not coherent with those from the study in Iran, which have shown that higher starchy food such as potato consumption was negatively associated with GDM risk, and there was no significant association with the consumption of total starchy or other starchy vegetables with GDM [30]. This possibly due to the confounding factors in their study did not take into account nutritional supplement. Additionally, there was a wider disparity in the diet characterization when compared to most studies on diet and pregnancy outcomes [9–11, 25, 26]. And the extraction of rice/flour products with proven healthy food [9–11], or with proven non-healthy food [25, 26] as a dietary structure may mask or exaggerate the effect.

Some researchers have considered the potential that high starchy food intake may influence the metabolic system [25, 31]. The possible reasons may be ascribed to the cereal and extensive processing of the starchy foods used in daily life that may result in the lack of dietary fibers, minerals and proteins in foods [32]. Moreover, these diets comprise fine grains that can be digested rapidly, thus increasing the dietary burden of the pancreas [25]. In addition, the refined grains and tubers have high glycemic index (GI) and glycemic load (GL) [32] that induce pathological glycemia and insulin resistance [33]. Other studies have revealed that the high GI group exhibited significantly higher risks of hyperlipidemia and metabolic disorders [31]. Besides, a previous research also confirmed that rice, as a principal component in the ‘Traditional’ diet pattern, has been positively associated with abnormal high-density lipoprotein in Chinese adults [34]. Larger cohort studies are required to examine this association in detail and to validate the findings of this study. It is beneficial to provide evidence on periconceptional preventative interventions to reduce the burden of adverse pregnancy outcomes.

### Strengths and limitations

Although the present study exhibited a small sample size, our case-to-control ratio reached a value of 1:4, which is in accordance with the highest statistical efficiency of case-control requirements. There was enough power with the nested case–control design to detect an odds ratio of 3 for the adverse pregnancy outcomes. In addition, smaller selection bias and information bias were the consequences of the collection of the exposure data prior to disease diagnosis. Moreover, additional comparable cases and controls were used in the present study as a result of the use of the same cohort. Because this was not a randomized controlled trial, we cannot rule out the possibility that residual confounding may be contributing to this apparent association. There may be unmeasured confounders resulting in the apparent positive relationship between high starchy foods taking in the first trimester and adverse pregnancy outcomes. However, we have adjusted for major factors known to confound this relationship. Nutritional supplementation was an important confounding factor. In this study, folic acid and folic acid-related multivitamins were adjusted in multivariate analysis, which was the main supplementary nutrients in periconceptional period [35, 36]. Besides, by focusing on diagnosis made after collection of the FFQ data we attempted to avoid bias due to changes in diet after diagnosis, which strengthened the reliability of our results.

One of the limitations of this study was the use of an unquantified food frequency questionnaire (FFQ) without any portion size information. As thus, the accuracy of derived food item information was lower than that of the gold standard method for collecting weighed dietary records. However, when studying the relationship between diet and disease, the use of dietary patterns has advantages over the usual methods of examining a single food [37]. And the results of previous studies comparing the use of factor analysis using FFQ with the weighed dietary records suggested that the two methods were comparable in the efficient when investigating the diet-disease associations [38, 39].

## Conclusion

High intake of starchy foods (characterized by high intake in noodle and flour products and/or rice and its products) during the first trimester of pregnancy may associated with increased risk for adverse pregnancy outcomes. Our work highlights the importance of promoting a healthy diet during pregnancy and suggests that behavior-change strategies may be necessary to improve perinatal outcomes and the health of the fetuses.

## Supplementary information

**Additional file 1.** Questionnaire in English version.

**Additional file 2: Figure S1.** Flow chart of participants in the study.

**Additional file 3: Table S1.** The rotation matrix of dietary patterns and factor loading distribution during the three months before pregnancy.

**Additional file 4: Table S2.** The rotation matrix of dietary patterns and factor loading distribution during the first trimester of pregnancy.

**Additional file 5: Table S3.** The association between six principal dietary patterns and adverse pregnancy outcomes.

## Data Availability

The datasets used and/or analyzed during the current study are available from the corresponding author on reasonable request.

## Abbreviations

aOR: Adjusted odds ratio
BMI: Body mass index
Cis: Confidence intervals
FFQ: Food-frequency questionnaire
GDM: Gestational diabetes mellitus
HDP: Hypertensive disorders in pregnancy
OR: Odds ratio
SGA: Small for gestational age
