# Supplementary material for: High starchy food intake may increase the risk of adverse pregnancy outcomes: a nested case-control study in the Shaanxi province of Northwestern China
Source: BMC Pregnancy Childbirth. 2019 Oct 21;19:362. doi: 10.1186/s12884-019-2524-z (PMC6802140; doi:10.1186/s12884-019-2524-z)
Supplement: Supplementary file 1 — Additional file 1. Questionnaire in English version. [file 12884_2019_2524_MOESM1_ESM.docx]

**Pre-pregnancy and prenatal examination project files in The First Affiliate Hospital of Xi’an Jiaoytong University**

**No. ___________ Date: _____________ Researcher: ____________**

**Name:** __________ [**Date**](javascript:;) [**of**](javascript:;) [**birth**](javascript:;): ________ **Age**: ________

**Phone number:** __________________

**Ethnic** Han Nationality Others_________

**Birth-place**: ____________________________

**Main place of residence during gestation:** ___________________________

**Occupation**: Famer Worker Administrative official Technician

Businessman or service staff [Individual](javascript:;) [household](javascript:;) or Private Enterprise

No job Others_________

**Degree of education**： Illiteracy Primary school Middle school High school Junior college Undergraduate Master degree or higher

**Current residence：** City Suburb Country

**Family** **Income(including husband’s income):**  ＜1000RMB 1000RMB-4000RMB 4000RMB-8000RMB 8000RMB-12000RMB ＞12000RMB Unknow

**---------------------------------------------------------------------------------------------------------------------------------**

**Husband**:

**Name:** __________ [**Date**](javascript:;) [**of**](javascript:;) [**birth**](javascript:;): ________ **Age:** ________

**Phone number**: __________________

**Nation:** Han Nationality others_________

**Occupation:** Famer Worker Administrative official Technician

Businessman or service staff [Individual](javascript:;) [household](javascript:;) or Private Enterprise

No job Others_________

**Degree of education：** Illiteracy Primary school Middle school High school Junior college Undergraduate Master degree or higher

**Consanguineous marriage:** Yes No

**---------------------------------------------------------------------------------------------------------------------------------**

**Menstrual history:**  **Age of menophania:** ______ **Menstrual period**:_____

**Menstrual cycle:** ______  **Regularity**: Regular Irregular

**Allergic history:** No Drugs_________ Food_____ Others________

**Are you currently pregnant:** Yes No

**If you are pregnant, the data of last menstrual period:** _______

**The way of fertilization:**  conceived naturally taking ovulation boosters

artificial insemination external fertilization

**Was the pregnancy planned or accidental:** planned accidental

**Weight before pregnancy**:________kg Hight:_____cm

**BMI:** _______ ＜18.5 18.5-24.0 24.0-28.0 ≥28.0

| **Food intake** | **Three months before pregnancy** | **First trimester** |
| --- | --- | --- |
| Rice and its products | ≥Once a day; 3-6 times a week; 1-2 times a week;2-3 times a month;≤Once a month; never or rarely | ≥Once a day; 3-6 times a week; 1-2 times a week;2-3 times a month;≤Once a month; never or rarely |
| Noodle and flour products | ≥Once a day; 3-6 times a week; 1-2 times a week;2-3 times a month;≤Once a month; never or rarely | ≥Once a day; 3-6 times a week; 1-2 times a week;2-3 times a month;≤Once a month; never or rarely |
| Fresh vegetables | ≥Once a day; 3-6 times a week; 1-2 times a week;2-3 times a month;≤Once a month; never or rarely | ≥Once a day; 3-6 times a week; 1-2 times a week;2-3 times a month;≤Once a month; never or rarely |
| Fresh fruit | ≥Once a day; 3-6 times a week; 1-2 times a week;2-3 times a month;≤Once a month; never or rarely | ≥Once a day; 3-6 times a week; 1-2 times a week;2-3 times a month;≤Once a month; never or rarely |
| Beef and mutton | ≥Once a day; 3-6 times a week; 1-2 times a week;2-3 times a month;≤Once a month; never or rarely | ≥Once a day; 3-6 times a week; 1-2 times a week;2-3 times a month;≤Once a month; never or rarely |
| Pork | ≥Once a day; 3-6 times a week; 1-2 times a week;2-3 times a month;≤Once a month; never or rarely | ≥Once a day; 3-6 times a week; 1-2 times a week;2-3 times a month;≤Once a month; never or rarely |
| Poultry meat | ≥Once a day; 3-6 times a week; 1-2 times a week;2-3 times a month;≤Once a month; never or rarely | ≥Once a day; 3-6 times a week; 1-2 times a week;2-3 times a month;≤Once a month; never or rarely |
| Fish, shrimp and other aquatic products | ≥Once a day; 3-6 times a week; 1-2 times a week;2-3 times a month;≤Once a month; never or rarely | ≥Once a day; 3-6 times a week; 1-2 times a week;2-3 times a month;≤Once a month; never or rarely |
| Eggs | ≥Once a day; 3-6 times a week; 1-2 times a week;2-3 times a month;≤Once a month; never or rarely | ≥Once a day; 3-6 times a week; 1-2 times a week;2-3 times a month;≤Once a month; never or rarely |
| Milk and its products | ≥Once a day; 3-6 times a week; 1-2 times a week;2-3 times a month;≤Once a month; never or rarely | ≥Once a day; 3-6 times a week; 1-2 times a week;2-3 times a month;≤Once a month; never or rarely |
| Beans and their products | ≥Once a day; 3-6 times a week; 1-2 times a week;2-3 times a month;≤Once a month; never or rarely | ≥Once a day; 3-6 times a week; 1-2 times a week;2-3 times a month;≤Once a month; never or rarely |
| Nuts | ≥Once a day; 3-6 times a week; 1-2 times a week;2-3 times a month;≤Once a month; never or rarely | ≥Once a day; 3-6 times a week; 1-2 times a week;2-3 times a month;≤Once a month; never or rarely |
| Fried food | ≥Once a day; 3-6 times a week; 1-2 times a week;2-3 times a month;≤Once a month; never or rarely | ≥Once a day; 3-6 times a week; 1-2 times a week;2-3 times a month;≤Once a month; never or rarely |
| Pickles and Chinese sauerkraut | ≥Once a day; 3-6 times a week; 1-2 times a week;2-3 times a month;≤Once a month; never or rarely | ≥Once a day; 3-6 times a week; 1-2 times a week;2-3 times a month;≤Once a month; never or rarely |
| Animal giblets | ≥Once a day; 3-6 times a week; 1-2 times a week;2-3 times a month;≤Once a month; never or rarely | ≥Once a day; 3-6 times a week; 1-2 times a week;2-3 times a month;≤Once a month; never or rarely |
| Onions or garlic | ≥Once a day; 3-6 times a week; 1-2 times a week;2-3 times a month;≤Once a month; never or rarely | ≥Once a day; 3-6 times a week; 1-2 times a week;2-3 times a month;≤Once a month; never or rarely |
| Green tea | ≥Once a day; 3-6 times a week; 1-2 times a week;2-3 times a month;≤Once a month; never or rarely | ≥Once a day; 3-6 times a week; 1-2 times a week;2-3 times a month;≤Once a month; never or rarely |
| Coffee | ≥Once a day; 3-6 times a week; 1-2 times a week;2-3 times a month;≤Once a month; never or rarely | ≥Once a day; 3-6 times a week; 1-2 times a week;2-3 times a month;≤Once a month; never or rarely |
| cola | ≥Once a day; 3-6 times a week; 1-2 times a week;2-3 times a month;≤Once a month; never or rarely | ≥Once a day; 3-6 times a week; 1-2 times a week;2-3 times a month;≤Once a month; never or rarely |

| **Nutritional supplements** | **Three months before pregnancy** | **First trimester** |
| --- | --- | --- |
| **Folic acid tablet** |  |  |
| Intaking dosage(mg/day) |  |  |
| Intaking duration(day) |  |  |
| **Multivitamins including folic acid** |  |  |
| Drug names |  |  |
| Intaking dosage(mg/day) |  |  |
| Intaking duration(day) |  |  |
