# Supplementary figures and images for: High starchy food intake may increase the risk of adverse pregnancy outcomes: a nested case-control study in the Shaanxi province of Northwestern China
Source: BMC Pregnancy Childbirth. 2019 Oct 21;19:362. doi: 10.1186/s12884-019-2524-z (PMC6802140; doi:10.1186/s12884-019-2524-z)

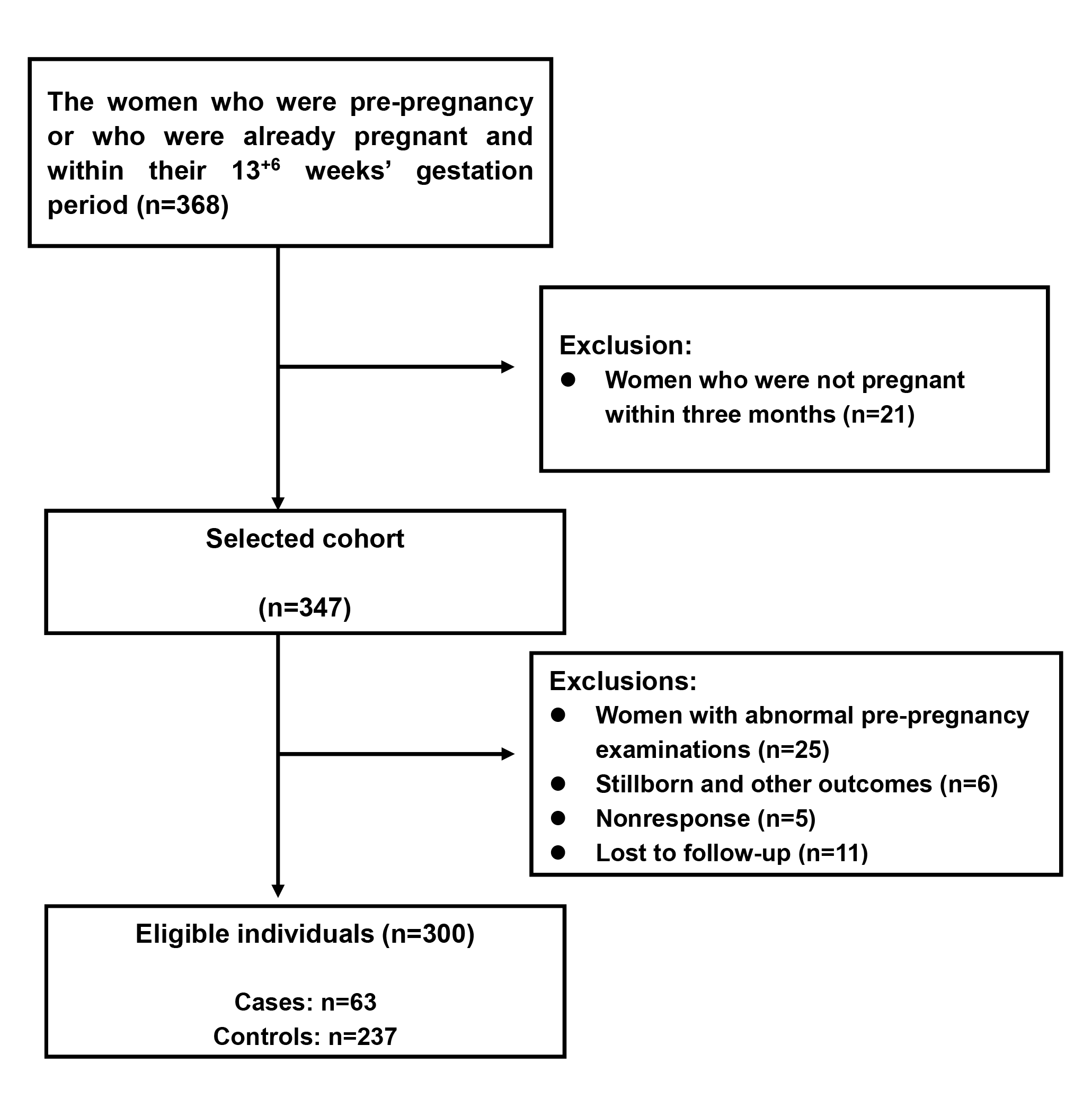

Supplement: Supplementary file 2 — Additional file 2: Figure S1. Flow chart of participants in the study. [file 12884_2019_2524_MOESM2_ESM.tif]
