# Supplementary material for: High starchy food intake may increase the risk of adverse pregnancy outcomes: a nested case-control study in the Shaanxi province of Northwestern China
Source: BMC Pregnancy Childbirth. 2019 Oct 21;19:362. doi: 10.1186/s12884-019-2524-z (PMC6802140; doi:10.1186/s12884-019-2524-z)
Supplement: Supplementary file 3 — Additional file 3: Table S1. The rotation matrix of dietary patterns and factor loading distribution during the three months before pregnancy. [file 12884_2019_2524_MOESM3_ESM.docx]

**Table S1 The rotation matrix of dietary patterns and factor loading distribution during the three months before pregnancy**

| **Food items** | **Factor loading** | | | | | | |
| --- | --- | --- | --- | --- | --- | --- | --- |
|  | **animal protein** | **Caffeine** | **healthy** | **processed** | | **starchy foods** | **vegetarian** |
| poultry | 0.695 | 0.256 |  |  |  | |  |
| beef and mutton | 0.677 | 0.153 | 0.237 |  |  | |  |
| fish and shrimp | 0.663 |  | 0.247 |  |  | |  |
| pork | 0.610 |  |  | 0.221 | 0.224 | | 0.115 |
| green tea | 0.103 | 0.795 |  | 0.158 |  | |  |
| coffee |  | 0.763 |  |  |  | |  |
| cola |  | 0.678 | -0.115 |  |  | |  |
| beans and their products |  |  | 0.726 | 0.103 | 0.138 | | -0.155 |
| milk and its products |  | 0.181 | 0.674 | -0.114 | -0.117 | | 0.290 |
| nuts | 0.286 | -0.212 | 0.559 |  |  | | 0.143 |
| eggs | 0.239 |  | 0.550 | 0.114 |  | |  |
| pickles/sauerkraut | -0.125 | 0.148 |  | 0.753 |  | |  |
| garlic |  |  | 0.199 | 0.579 | 0.248 | | 0.147 |
| animal giblets | 0.363 |  |  | 0.550 | -0.118 | | -0.294 |
| fried food | 0.173 | 0.179 |  | 0.516 | -0.360 | | 0.124 |
| noodle and flour products | -0.125 |  | 0.142 |  | 0.692 | | -0.206 |
| rice and its products | 0.129 |  |  |  | 0.644 | | 0.146 |
| fresh vegetables | 0.196 |  |  |  | 0.478 | | 0.451 |
| fresh fruits |  |  | 0.118 | 0.123 |  | | 0.823 |
