# Supplementary material for: High starchy food intake may increase the risk of adverse pregnancy outcomes: a nested case-control study in the Shaanxi province of Northwestern China
Source: BMC Pregnancy Childbirth. 2019 Oct 21;19:362. doi: 10.1186/s12884-019-2524-z (PMC6802140; doi:10.1186/s12884-019-2524-z)
Supplement: Supplementary file 4 — Additional file 4: Table S2. The rotation matrix of dietary patterns and factor loading distribution during the first trimester of pregnancy. [file 12884_2019_2524_MOESM4_ESM.docx]

**Table S2 The rotation matrix of dietary patterns and** **factor loading distribution during the first trimester of pregnancy**

| **Food items** | **Factor loading** | | | | | |
| --- | --- | --- | --- | --- | --- | --- |
|  | **animal protein** | **caffeine** | **healthy** | **processed** | **starchy foods** | **vegetarian** |
| poultry | 0.733 | 0.186 |  |  |  |  |
| beef and mutton | 0.699 | 0.140 |  | 0.117 |  |  |
| fish and shrimp | 0.657 | -0.153 | 0.155 |  | 0.178 |  |
| pork | 0.589 |  | 0.158 | 0.207 | -0.100 |  |
| green tea |  | 0.840 |  |  |  |  |
| coffee |  | 0.756 |  |  | -0.141 |  |
| cola | 0.182 | 0.699 |  |  | 0.119 |  |
| beans and their products |  | 0.156 | 0.770 | -0.147 | 0.169 |  |
| milk and its products | 0.177 |  | 0.648 |  | -0.234 |  |
| nuts |  |  | 0.611 | 0.241 | 0.134 | 0.131 |
| eggs | 0.392 |  | 0.513 |  |  |  |
| pickles/sauerkraut | -0.102 |  |  | 0.676 |  |  |
| garlic |  |  |  | 0.656 | 0.150 | -0.123 |
| animal giblets | 0.293 |  |  | 0.601 |  | 0.120 |
| fried food | 0.238 |  |  | 0.562 | 0.144 | 0.178 |
| noodle and flour products |  |  |  |  | 0.737 |  |
| rice and its products |  |  |  |  | 0.729 | 0.121 |
| fresh vegetables |  |  | 0.100 |  | -0.148 | 0.817 |
| fresh fruits | 0.103 |  |  |  | 0.291 | 0.726 |
