# Supplementary material for: High starchy food intake may increase the risk of adverse pregnancy outcomes: a nested case-control study in the Shaanxi province of Northwestern China
Source: BMC Pregnancy Childbirth. 2019 Oct 21;19:362. doi: 10.1186/s12884-019-2524-z (PMC6802140; doi:10.1186/s12884-019-2524-z)
Supplement: Supplementary file 5 — Additional file 5: Table S3. The association between six principal dietary patterns and adverse pregnancy outcomes. [file 12884_2019_2524_MOESM5_ESM.docx]

**Table S3 The association between six principal dietary patterns and adverse pregnancy outcomes**

| **dietary patterns** | **Overall adverse pregnancy outcomes** | | |  | **Single adverse pregnancy outcomes** | | |  | **Multiple adverse pregnancy outcomes** | | |
| --- | --- | --- | --- | --- | --- | --- | --- | --- | --- | --- | --- |
|  | **N (%)** | **OR(95%CI)** | **OR(95%CI)** **^a^** |  | **N (%)** | **OR(95%CI)** | **OR(95%CI) ^a^** |  | **N (%)** | **OR(95%CI)** | **OR(95%CI)** **^a^** |
| **Three months before pregnancy** | | | | | | | | | | | |
| Animal protein | |  |  |  |  |  |  |  |  |  |  |
| low intake | 31(21.2) | 1.000(0.571,1.752) | 0.995(0.521, 1.747) |  | 15(11.5) | 0.789(0.383, 1.629) | 0.755(0.323, 1.642) |  | 16(12.2) | 1.333(0.604, 2.943) | 1.211(0.525, 3.013) |
| high intake | 31(21.2) | 1.000 | 1.000 |  | 19(14.2) | 1.000 | 1.000 |  | 12(9.4) | 1.000 | 1.000 |
| Caffeine |  |  |  |  |  |  |  |  |  |  |  |
| low intake | 27(18.5) | 1.000 | 1.000 |  | 16(11.9) | 1.000 | 1.000 |  | 11(8.5) | 1.000 | 1.000 |
| high intake | 35(24.0) | 1.390(0.790, 2.444) | 1.603(0.881, 2.942) |  | 18(14.0) | 1.206(0.586, 2.481) | 1.521(0.792, 3.254) |  | 17(13.3) | 1.657(0.743, 3.692) | 1.632(0.671, 3.952) |
| Healthy |  |  |  |  |  |  |  |  |  |  |  |
| low intake | 33(22.6) | 1.178(0.672, 2.067) | 1.162(0.622, 2.145) |  | 17(13.1) | 1.035(0.504, 2.128) | 1.047(0.512, 2.242) |  | 16(12.4) | 1.381(0.625, 3.048) | 1.364(0.556, 3.387) |
| high intake | 29(19.9) | 1.000 | 1.000 |  | 17(12.7) | 1.000 | 1.000 |  | 12(9.3) | 1.000 | 1.000 |
| Processed |  |  |  |  |  |  |  |  |  |  |  |
| low intake | 36(24.7) | 1.000 | 1.000 |  | 18(14.1) | 1.000 | 1.000 |  | 18(14.1) | 1.000 | 1.000 |
| high intake | 26(17.8) | 0.662(0.376, 1.167) | 0.643(0.352, 1.171) |  | 16(11.8) | 0.815(0.396, 1.676) | 0.733(0.325, 1.564) |  | 10(7.7) | 0.509(0.225, 1.151) | 0.703(0.263, 1.574) |
| Starchy food |  |  |  |  |  |  |  |  |  |  |  |
| low intake | 30(20.5) | 1.000 | 1.000 |  | 14(10.8) | 1.000 | 1.000 |  | 16(12.1) | 1.000 | 1.000 |
| high intake | 32(21.9) | 1.085(0.619, 1.902) | 1.121(0.712, 2.123) |  | 20(14.9) | 1.454(0.700, 3.017) | 1.473(0.682, 3.234) |  | 12(9.5) | 0.763(0.346, 1.685) | 0.728(0.295, 1.728) |
| Vegetarian |  |  |  |  |  |  |  |  |  |  |  |
| low intake | 30(20.5) | 0.921(0.526, 1.615) | 1.084(0.533, 1.716) |  | 17(12.8) | 0.983(0.478, 2.020) | 1.177(0.639, 2.602) |  | 13(10.1) | 0.852(0.388, 1.870) | 0.892(0.343, 2.081) |
| high intake | 32(21.9) | 1.000 | 1.000 |  | 17(13.0) | 1.000 | 1.000 |  | 15(11.6) | 1.000 | 1.000 |
| **The first trimester of pregnancy ^b^** | | | | | | | | | | | |
| Animal protein |  |  |  |  |  |  |  |  |  |  |  |
| low intake | 29(19.9) | 0.849(0.484, 1.489) | 0.841(0.436, 1.571) |  | 15(11.4) | 0.762(0.369, 1.574) | 0.671(0.301, 1.502) |  | 14(10.7) | 0.966(0.441, 2.117) | 1.019(0.418, 2.483) |
| high intake | 33(22.6) | 1.000 | 1.000 |  | 19(14.4) | 1.000 | 1.000 |  | 14(11.0) | 1.000 | 1.000 |
| Caffeine |  |  |  |  |  |  |  |  |  |  |  |
| low intake | 33(22.6) | 1.000 | 1.000 |  | 16(12.4) | 1.000 | 1.000 |  | 17(13.1) | 1.000 | 1.000 |
| high intake | 29(19.9) | 0.849(0.484, 1.489) | 0.931(0.495, 1.838) |  | 18(13.3) | 1.087(0.528, 2.235) | 1.356(0.571, 2.825) |  | 11(8.6) | 0.625(0.280, 1.393) | 0.608(0.235, 1.576) |
| Processed |  |  |  |  |  |  |  |  |  |  |  |
| low intake | 33(22.6) | 1.000 | 1.000 |  | 19(14.4) | 1.000 | 1.000 |  | 14(11.0) | 1.000 | 1.000 |
| high intake | 29(19.9) | 0.849(0.484, 1.489) | 0.801(0.424, 1.462) |  | 15(11.4) | 0.762(0.369, 1.574) | 0.742(0.330, 1.671) |  | 14(10.7) | 0.966(0.441, 2.117) | 1.002(0.512,2.443) |
| Healthy |  |  |  |  |  |  |  |  |  |  |  |
| Low intake | 34(23.3) | 1.279(0.729, 2.247) | 1.264(0.680, 2.443) |  | 16(12.5) | 0.937(0.455, 1.927) | 1.112(0.481, 2.322) |  | 18(13.8) | 1.896(0.839, 4.285) | 1.906(0.827,4.551) |
| high intake | 28(19.2) | 1.000 | 1.000 |  | 18(13.2) | 1.000 | 1.000 |  | 10(7.8) | 1.000 | 1.000 |
| Starchy food |  |  |  |  |  |  |  |  |  |  |  |
| low intake | 21(14.4) | 1.000 | 1.000 |  | 9(6.7) | 1.000 | 1.000 |  | 12(8.8) | 1.000 | 1.000 |
| high intake | 41(28.1) | 2.324(1.293, 4.178)* | 2.337(1.253, 4.331)* |  | 25(19.2) | 3.307(1.479,7.395)* | 3.321(1.373, 7.250)* |  | 16(13.2) | 1.587(0.719, 3.505) | 1.448(0.660, 3.265) |
| Vegetarian |  |  |  |  |  |  |  |  |  |  |  |
| low intake | 27(18.5) | 0.720(0.409, 1.266) | 0.720(0.387, 1.336) |  | 16(11.9) | 0.829(0.403, 1.706) | 0.822(0.381, 1.800) |  | 11(8.5) | 0.604(0.271, 1.345) | 0.593(0.337, 1.532) |
| high intake | 35(24.0) | 1.000 | 1.000 |  | 18(14.0) | 1.000 | 1.000 |  | 17(13.3) | 1.000 | 1.000 |

^a^ Multivariate analysis and adjustment of age, residence, alcohol intake, economic situation, smoking and pre-pregnancy BMI;

^b^ For the first trimester of pregnancy, ORs were additionally adjusted the nutrient supplementation；

* *p* < 0.05
